# The Human Adenovirus Type 5 E1B 55 kDa Protein Obstructs Inhibition of Viral Replication by Type I Interferon in Normal Human Cells

**DOI:** 10.1371/journal.ppat.1002853

**Published:** 2012-08-09

**Authors:** Jasdave S. Chahal, Ji Qi, S. J. Flint

**Affiliations:** Princeton University, Department of Molecular Biology, Lewis Thomas Laboratory, Princeton, New Jersey, United States of America; University of Michigan, United States of America

## Abstract

Vectors derived from human adenovirus type 5, which typically lack the E1A and E1B genes, induce robust innate immune responses that limit their therapeutic efficacy. We reported previously that the E1B 55 kDa protein inhibits expression of a set of cellular genes that is highly enriched for those associated with anti-viral defense and immune responses, and includes many interferon-sensitive genes. The sensitivity of replication of E1B 55 kDa null-mutants to exogenous interferon (IFN) was therefore examined in normal human fibroblasts and respiratory epithelial cells. Yields of the mutants were reduced at least 500-fold, compared to only 5-fold, for wild-type (WT) virus replication. To investigate the mechanistic basis of such inhibition, the accumulation of viral early proteins and genomes was compared by immunoblotting and qPCR, respectively, in WT- and mutant-infected cells in the absence or presence of exogenous IFN. Both the concentration of viral genomes detected during the late phase and the numbers of viral replication centers formed were strongly reduced in IFN-treated cells in the absence of the E1B protein, despite production of similar quantities of viral replication proteins. These defects could not be attributed to degradation of entering viral genomes, induction of apoptosis, or failure to reorganize components of PML nuclear bodies. Nor was assembly of the E1B- and E4 Orf6 protein- E3 ubiquitin ligase required to prevent inhibition of viral replication by IFN. However, by using RT-PCR, the E1B 55 kDa protein was demonstrated to be a potent repressor of expression of IFN-inducible genes in IFN-treated cells. We propose that a primary function of the previously described transcriptional repression activity of the E1B 55 kDa protein is to block expression of IFN- inducible genes, and hence to facilitate formation of viral replication centers and genome replication.

## Author Summary

The most frequently used therapeutic vectors for gene transfer or cancer treatment are derived from human adenovirus type 5 (Ad5). We have observed previously that the E1B 55 kDa protein encoded by a gene routinely deleted from these vectors represses expression of numerous cellular genes regulated by interferon (IFN) α and β, which are important components of the innate immune response to viral infection. We therefore compared synthesis of pre-mRNA from IFN-inducible genes, viral yields and early reactions in the infectious cycle in normal human cells exposed to exogenous IFN and infected by wild-type or E1B 55 kDa null-mutant viruses. We report that the E1B 55 kDa protein is a potent repressor of expression of IFN-regulated genes, and protects viral replication against anti-viral actions of IFN by blocking inhibition of formation of viral replication centers and genome replication. These observations provide the first information about the function of the transcription repression activity of E1B during the infectious cycle. Importantly, they also suggest new design considerations for adenoviral vectors that can circumvent induction of innate immune responses, currently a major therapeutic limitation.

## Introduction

A major obstacle to the therapeutic application and efficacy of adenoviral vectors is the induction of powerful innate and pro-inflammatory immune responses following systemic delivery [1]–[4], independently of viral gene expression [5]–[9]. The constellations of chemokines produced in response to adenovirus vector infection depend on the host cell type and its species of origin, as do the mechanisms by which infection is detected by host cell pattern recognition receptors to activate signal transduction pathways and transcription of genes that encode these immunomodulators [2]–[4]. Nevertheless, production of several chemokines, including Rantes, Mip1-α and IL-8, and such cytokines as interferon (IFN) α and β, Tnf-α and IL-6 has been observed upon infection of a wide variety of established and primary human and murine cells in culture and *in vivo* [4], [7]–[19]. Interferon α and β, designated hereafter IFN, bind to the same heterodimeric receptor to establish a front line of anti-viral defense via stimulation of transcription of numerous genes [20]–[23]. The products of such interferon-stimulated genes (ISGs) inhibit replication of a wide variety of viruses by multiple direct or indirect mechanisms [22], [24]–[27]. Proteins encoded by ISGs also reinforce synthesis of IFN and other cytokines, promote processing and presentation of antigens, and modulate the activity of important effector cells of the immune system [22], [25]–[29].

The replication of human adenovirus type 5 (Ad5), from which nearly all vectors have been derived, is refractory to IFN in several lines of established human cells [30]–[32], as a result of the actions of several viral gene products that counter the effects of the cytokine. The first to be identified, the small viral RNA, VA-RNA I [30] binds to, and prevents activation of, the interferon-induced, double-stranded RNA-activated protein kinase, which phosphorylates elF2-α to inhibit translation during the late phase of infection [33]. More recently, it has been established that the viral E4 Orf3 protein is required to prevent inhibition of viral DNA synthesis in type I IFN-treated cells [32]. This function of the E4 Orf3 protein correlates with reorganization of the promyelocytic leukemia protein (Pml) from the discrete, nuclear Pml bodies present in uninfected cells to track-like structure that also contain the viral protein, and is abrogated by shRNA-mediated knockdown of Pml or Daxx [34]. In addition, the viral E1A proteins suppress transcription of interferon-sensitive genes in infected cells [35]–[39] and both block activation of the Jak-Stat signaling pathway that induces transcription of ISGs and interact directly with Stat 1 co-activators [37]–[43]. The contributions of these viral products to modulation of innate immune responses *in vivo* have not been investigated intensively. Nevertheless, both the 243R E1A protein and the E3 gene, which encodes several proteins that inhibit inflammatory responses and apoptosis induced by binding of their ligands to the Tnfα and related receptors [44], [45], have been shown to decrease such responses to adenoviral vectors in various murine organs or tissues [46]–[49]. Comparison of induction of edema in mouse ears by vectors carrying different combinations of E1A, E1B and E3 coding sequences also implicated the E1B 19 kDa and 55 kDa proteins in inhibition of inflammatory responses [49]. The anti-inflammatory activity of the E1B 19 kDa protein was proposed to be the result of the anti-apoptotic activity of this viral Bcl-2 homologue [50], [51].

The E1B 55 kDa protein makes an important contribution to optimizing the host cell environment for efficient viral replication [52], [53] via formation of a virus-specific E3 ubiquitin ligase that also contains the viral E4 Orf6 protein, Cul5 and several other cellular proteins [54], [55]. The activity of this enzyme targets the cellular proteins p53, Mre11, Rad50, DNA ligase IV and integrin α3 for proteasomal degradation [54]–[60]. The destruction of Mre11 and Rad50 facilitates inhibition of the DNA double stranded break repair response and helps circumvent inhibition of viral DNA in infected cells [61]–[63], while degradation of DNA ligase IV contributes to prevention of genome concatamerization [59]. Assembly of the virus-specific E3 ubiquitin ligase is also necessary for induction of selective export from the nucleus of viral late mRNAs [64], [65].

One of the earliest functions ascribed to the E1B 55 kDa protein was repression of transcription of genes regulated by the tumor suppressor p53 in *in vitro* and transient expression assays [66], [67]. This activity correlates with the ability of the E1B protein to cooperate with E1A proteins to transform rodent cells [68]–[71]. It has long been supposed that inhibition of p53-dependent transcription by the E1B 55 kDa protein in infected cells would contribute to preventing induction of cell cycle arrest or apoptosis upon stabilization and activation of p53 by the viral E1A proteins (e.g. [52], [72], [73]). However, when p53 accumulates to high concentrations in cells infected by Ad5 mutants that cannot direct synthesis of this E1B protein (E1B 55 kDa null-mutants), expression of p53-activated genes is not increased [74]–[76]. Indeed, as assessed by microarray hybridization, the reversal of the p53 transcriptional program is as complete in normal human cells infected by an E1B 55 kDa-null-mutant as in wild-type Ad5-infected cells [76]. However, in the absence of the E1B protein, expression of some 340 genes highly enriched for those associated with immune responses and anti-viral defense was increased significantly [76]. In particular, we observed that this set contained many interferon-sensitive genes, including *GBP1-5, IF1H1 (MDA5), IF1T2, MX2* and *TAP1* [76]. These observations suggested that repression of expression of such genes by the E1B 55 kDa protein might protect Ad5-infected cells against anti-viral measures induced by type I IFN. We now report the results of experiments designed to test this hypothesis, which demonstrate that the E1B 55 kDa protein represses expression of ISGs and blocks type I IFN-induced inhibition of viral DNA synthesis and replication in normal human cells.

## Results

### The E1B 55 kDa protein blocks inhibition of Ad5 replication by type I interferon in normal human cells

**Figure 1:**
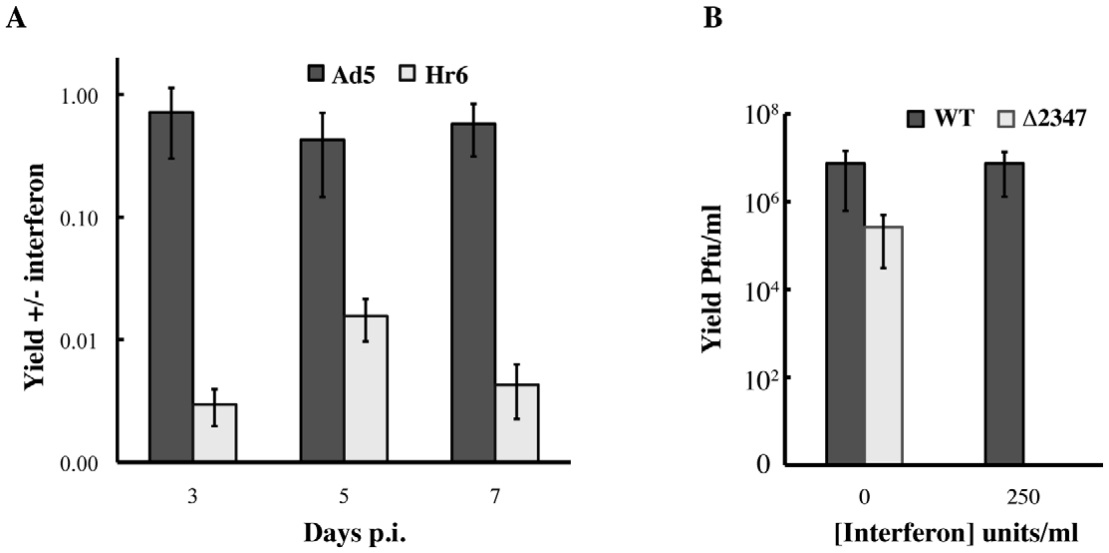
Effect of IFN treatment on wild type and E1B 55 kDa null-mutant virus replication in HFFs. (A) Viral yields were determined by plaque assay on 293 cells after infection of IFN-treated and untreated HFFs with 3 p.f.u./cell Ad5 or Hr6 for the periods indicated, and are shown as the ratios of these values. (B) Yields were determined 2 days after infection with 30 p.f.u./cell AdEasy or AdEasy E1Δ2347. The averages of two independent experiments and the standard deviations calculated as described in Materials and Methods are shown in both panels.

In initial experiments to investigate whether repression of expression of interferon-stimulated genes (ISGs) by the E1B 55 kDa protein protects Ad5-infected cells from the anti-viral defenses induced by this cytokine, replication of Ad5 and the E1B 55 kDa null-mutant Hr6 were compared in HFFs treated with exogenous IFN. Cells were maintained in the presence of 500 units/ml IFN, or of vehicle only control, for 24 hrs., prior to and during infection with 3 p.f.u./cell Ad5 or Hr6. They were harvested after increasing periods of infection, and viral yields measured by plaque assay on complementing 293 cells as described in Materials and Methods. This IFN treatment regimen decreased the yields of Ad5 by less than 3 fold (Figure 1A), in agreement with previous observations (see Introduction). In contrast, replication of Hr6 was reduced to a much greater degree, up to 500-fold.

The Hr6 mutant was isolated after nitrous acid mutagenesis of Ad5 by virtue of more efficient replication in complementing 293 cells [77], [78] than in non-complementing cells [79]. The mutation responsible for this phenotype was mapped to the E1B 55 kDa protein coding sequence by marker rescue and sequencing [80]. We have observed recently that the Hr6 genomes contain at least one additional mutation outside the E1B gene that substantially reduces the infectivity of virus particles (S. Kato, J. C. and S.J. F., manuscript in preparation). As described above, adenoviral VA-RNA I, E1A proteins and the E4 Orf3 protein have been reported previously to protect Ad5 replication against the inhibitory effects of IFN. It was therefore essential to determine whether the increased sensitivity of Hr6 replication to inhibition by IFN was the result of mutations in the E1B gene, or elsewhere in the genome. To this end, we exploited a mutant carrying the Hr6 E1B 55 kDa frameshift mutation (deletion of base-pair 2347) [80] introduced into the genome of a phenotypically wild-type, E1-containing derivative of AdEasy [81]. As reported elsewhere [82], no E1B 55 kDa protein can be detected in HFFs infected by this mutant (AdEasyE1Δ2347), and, as expected in the absence of this viral protein [80], [83]–[86], expression of viral late genes was impaired. HFFs maintained in the absence or presence of IFN were infected with 30 p.f.u./cell AdEasyE1Δ2347 or its parent AdEasyE1 [82], and yields determined after a single cycle of replication at 2 days p. i. Consistent with the results described above, replication of AdEasyE1 was inhibited by only a modest degree (<4-fold) in IFN treated cells (Figure 1B). However, in the presence of IFN, the yield of AdEasyE1Δ2347 was inhibited nearly 300-fold (Figure 1B), indicating that E1B 55 kDa protein prevents IFN-induced inhibition of replication in HFFs.

**Figure 2:**
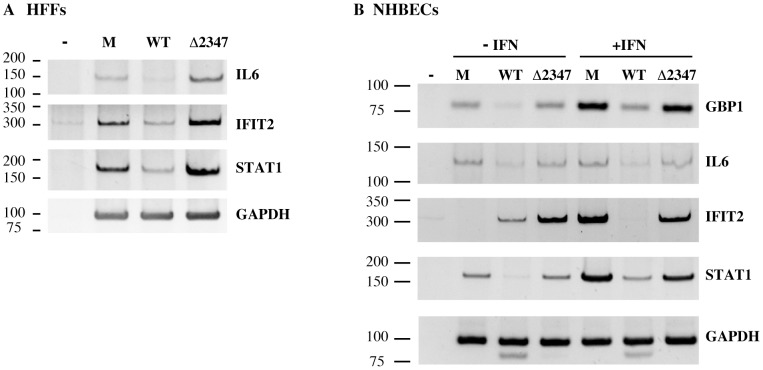
Comparison of the steady state concentrations of IFN-inducible RNAs in wild-type -and E1B 55 kDa null-mutant-infected cells. (A) The concentrations of the primary transcripts of the IFN-inducible genes indicated, and of GADPH mRNA, were compared by RT-PCR as described in Materials and Methods in untreated HFFs infected with 30 p.f.u./cell AdEasy E1 (WT) or AdEasyE1Δ2347 (Δ2347), or mock-infected (M). (B) The primary transcripts and mRNAs indicated were examined in NHBECs infected for 24 hrs with the viruses indicated or mock-infected (M), and untreated or exposed to IFN as indicated. In both panels, the positions of DNA markers (bp) are indicted at the left, and samples marked – above the lanes are the products of reactions that contained Δ2347-infected cell RNA, but no reverse transcriptase.

As described in the Introduction, it has been reported previously that the E1B 55 kDa protein can repress transcription in simplified experimental systems. This property suggested that this protein is likely to inhibit transcription of ISGs in infected cells, but the microarray hybridization data collected previously [76] cannot distinguish among the multiple mechanisms by which RNA concentrations can be regulated. The concentrations of pre-mRNAs of representative ISGs increased in expression in Hr6- compared to wild type-infected HFFs [76] were therefore examined in the presence and absence of the E1B protein. HFFs that were not exposed to IFN were infected with 30 p.f.u./cell AdEasyE1 or AdEasyE1Δ2347 for 30 hrs, and primary transcripts detected by using reverse transcription with random priming, followed by PCR with primers specific for three ISG pre-mRNAs, that is, spanning exon-intron junctions (see Materials and Methods). To provide an internal control, GAPDH mRNA was examined in parallel. Pre-mRNAs transcribed from the *IL6, IFIT2* and *STAT1* genes were present in mock-infected cells, and decreased significantly in concentration following infection with AdEasyE1, whereas only minimal differences in *GAPDH* mRNA were detected (Figure 2A). In contrast, synthesis of these ISG pre-mRNAs was not repressed in AdEasyE1Δ2347-infected HFFs, but rather these RNAs accumulated to higher concentrations than observed in uninfected or wild type-infected cells (Figure 2A). For example, quantification of signals as described in Materials and Methods indicated that the concentration of *IL6* pre-mRNA was 14-fold higher in mutant compared to wild-type-infected cells, whereas that of *GAPDH* mRNA was only 1.2-fold greater. These observations, which are consistent with our microarray hybridization data [76], indicate that the E1B 55 kDa protein is a potent repressor of ISG pre-mRNA synthesis in infected cells.

Although HFFs are permissive for adenovirus replication in tissue culture, we wished to confirm the sensitivity to IFN of E1B 55 kDa null-mutants in normal human bronchial/tracheal epithelial cells (NHBECs), which better represent the host cell type encountered by serotype C adenoviruses in their natural site of infection, the upper respiratory tract [87]. To investigate the sensitivity of NHBECs to IFN, the concentrations of ISG pre-mRNAs were compared before and after IFN-treatment. Because phenotypes exhibited by E1B 55 kDa-null-mutants of Ad5 have been reported to be cell-type dependent [83], [88]–[91], we also examined the effects of infection in the presence or absence of the E1B 55 kDa protein on ISG expression. NHBECs treated with IFN or control as described in Materials and Methods were infected with 30 p.f.u./cell AdEasyE1 or AdEasyE1Δ2347 for 24 hrs, and the concentrations of pre-mRNAs and of *GAPDH* mRNA examined by RT-PCR. As observed in HFFs (Figure 2A), *IL6* and *STAT1* pre-mRNAs were detected in uninfected, untreated NHBECs, and accumulated to reduced concentrations in AdEasyE1-, but not in AdEasyE1Δ2347-, infected cells (Figure 2B). The same pattern was observed for *GBP1* mRNA. In these cells, *IFIT2* pre-mRNA could be detected only following infection, and was present in significantly greater quantities in the absence of the E1B 55 kDa protein (Figure 2B). With the exception of *IL6*, the RNA products of these genes accumulated to increased concentrations in mock-infected cells exposed to IFN, indicating that NHBECs respond to this cytokine. Expression of all the ISGs examined was repressed in IFN-treated cells when infected by the wild-type virus, but not following AdEasyE1Δ2347 infection (Figure 2B).

**Figure 3:**
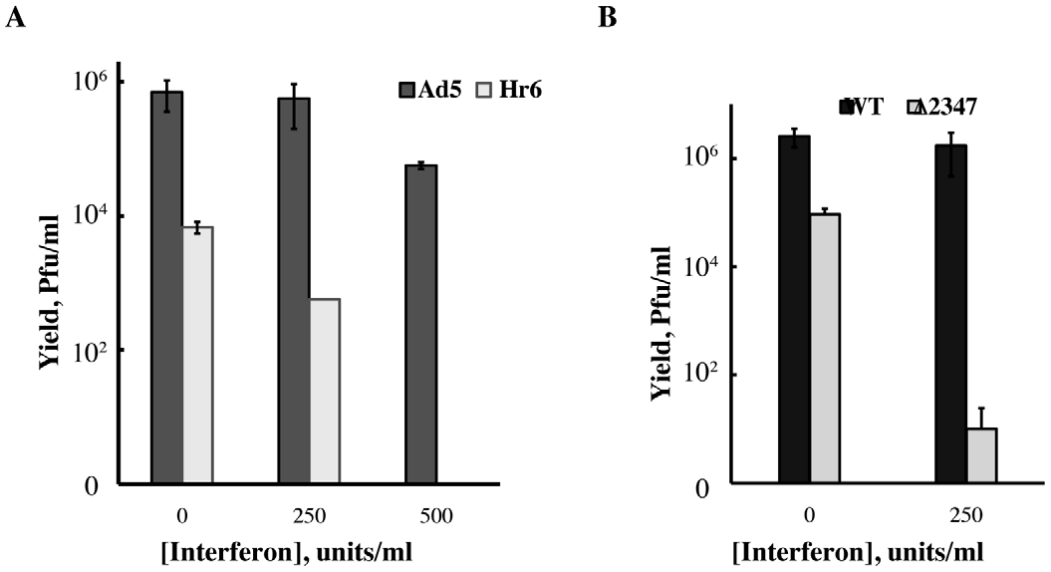
Effects of IFN treatment on wild type and E1B 55 kDa null-mutant virus replication in NHBECs. Viral yields were determined 36 hrs. after infection of untreated and IFN-treated NHBEs with 5 p.f.u./cell Ad5 or Hr6 (A) or with 5 p.f.u./cell AdEasyE1 (WT) or AdEasyE1Δ2347 (Δ2347) (B). In both panels, the averages of two independent experiments and the standard deviations calculated as described in Materials and Methods are shown.

To confirm that the sensitivity of E1B 55 kDa null-mutant virus replication to IFN was not specific to HFFs, the replication of the E1B 55 kDa-null-mutants Hr6 and AdEasyE1Δ2347 was compared to that of the corresponding wild-type virus in IFN-treated or untreated NHBECs. In these experiments, IFN had only a minor effect on replication of Ad5 or AdEasyE1 (Figures 3A and B). Treatment with 250 U/ml IFN reduced Hr6 and AdEasyE1Δ2347 titers by between two and three orders of magnitude (Figures 3A and 3B). Our previous studies have established that in these epithelial cells, in contrast to HFFs, E1B 55 kDa null-mutants do not exhibit defects in viral genome replication [92]. It is therefore unlikely that replication of the mutants would exhibit a lower degree of sensitivity to IFN at times later in the infectious cycle than examined in these experiments (36 hrs. p. i.). *In toto* these data indicate that exposure to exogenous IFN induces an antiviral state in HFFs and NHBECs that is detrimental to adenovirus replication, and blocked by the E1B 55 kDa protein.

### IFN pretreatment inhibits viral DNA synthesis in the absence of the E1B 55 kDa protein

**Figure 4:**
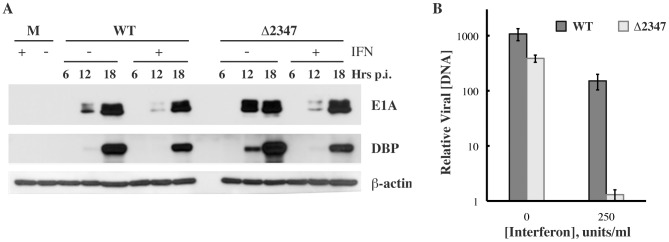
Effects of IFN on early reactions in the infectious cycle in the presence and absence of the E1B 55 kDa protein. (A) IFN treated and untreated NHBECs were infected with 5 p.f.u./cell AdEasyE1 (WT) or AdEasyEΔ23471 (Δ2347) and whole-cell lysates prepared after the periods indicated. The viral E1A proteins and DBP and cellular β-actin were examined by immunoblotting. (B) Nuclear DNA from IFN treated and untreated NHBECs infected with 5 p.f.u./cell AdEasyE1 (WT) or AdEasyE1Δ2347 (Δ2347) was isolated at 24 hrs. p. i. and the concentration of viral DNA measured by qPCR as described in Materials and Methods. The values shown were normalized relative to the input concentrations measured at 2 hrs. p. i., and represent the average and standard deviations calculated as described in Materials and Methods of two independent experiments.

It is well established that exposure of cells to type I IFN can restrict replication of many types of virus by multiple mechanisms, including inhibition of viral gene expression, mRNA translation, and genome replication [22], [24], [93], [94]. As a first step to identify the reaction(s) in the adenoviral life cycle that are protected by the E1B 55 kDa protein from inhibition induced by IFN signaling, the accumulation of viral early proteins was monitored in NHBECs infected with AdEasyE1 or AdEasyE1Δ2347. Cells were treated with IFN as described above, and harvested after increasing periods of infection. Whole cell lysates were prepared, and the steady-state concentrations of the E1A proteins and the E2 DNA-binding protein (DBP) examined by immunoblotting (Figure 4A). Somewhat reduced concentrations of E1A proteins were observed in IFN-treated compared to untreated cells at 12 hrs. p. i., but the quantities of these proteins detected in AdEasyE1- and AdEasyE1Δ2347-infected cells exposed to the cytokine were similar at both 12 and 18 hrs. p. i. (Figure 4A). The concentrations of DBP were reduced to a small degree by IFN-treatment of cells infected by AdEasyE1 or AdEasyE1Δ2347, by approximately 40% and 55%, respectively. These results indicate that type I IFN induced modest decreases in the steady-state concentrations of viral early proteins, including E2 replication proteins epitomized by the DBP, regardless of whether the E1B 55 kDa protein was synthesized in infected cells. We therefore examined the next reaction in the infectious cycle, synthesis of viral genomes.

**Figure 5:**
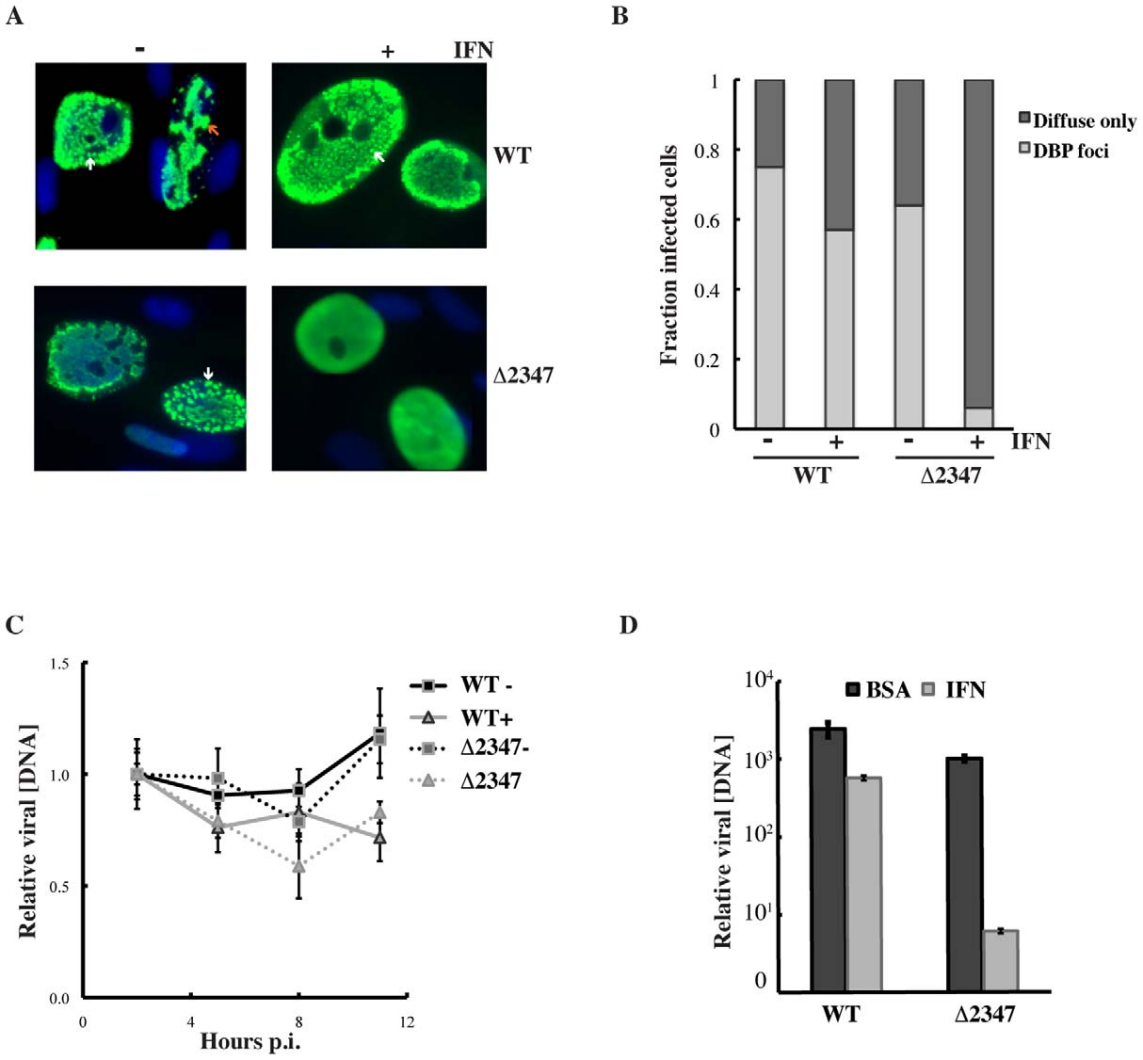
The E1B 55 kDa protein prevents inhibition of formation of viral replication centers in IFN-treated cells. (A) HFFs untreated or treated with IFN as indicted were infected with 30 p.f.u./cell AdEasyE1 (WT) or AdEasyE1Δ2347 (Δ2347), and the E2 DBP (green) examined by immunofluorescence as described in Materials and Methods. Nuclei were stained with DAP1 (blue). The white and orange arrows indicate discrete DBP foci, and larger structures, respectively. (B) The numbers of infected cells exhibiting replication centers or only diffuse nuclear DBP staining (light and dark gray bars, respectively) respectively were counted, and are represented as the fraction of the total number of infected cells (n = 161 and 151, WT - and +IFN respectively, and 151 for Δ2347 both – and +IFN). (C and D) IFN treated (+) and untreated (−) NHBECs were infected with 5 p.f.u./cell AdEasyE1(WT) or AdEasyE1Δ2347 (Δ2347) and nuclear concentrations determined at the early (C) and later (D) times after infection indicated. Values were corrected using the concentrations of β-actin internal control DNA, and normalized relative to the input concentrations at 2 hrs. p. i. The averages and standard deviations calculated as described in Materials and Methods of two independent experiments are shown.

NHBECs treated with IFN or untreated as described above were infected with 5 p.f.u./ml AdEasyE1 or AdEasyE1Δ2347 for 24 hrs. Nuclear DNA was isolated, and viral DNA concentration was measured by qPCR as described in Materials and Methods. The concentrations of the viral genome at 24 hrs. p. i., normalized to the input concentrations measured at 2 hrs. p. i., were observed to be reduced upon IFN pretreatment by approximately 7-fold in AdEasyE1-infected cells, but by nearly 300-fold in AdEasyE1Δ2347-infected cells (Figure 4B). We also monitored the formation of viral replication centers by using immunoflourescence to visualize DBP in IFN treated or untreated HFFs infected with the mutant or its wild-type parent, as described in Materials and Methods. The majority of untreated cells infected with AdEasy E1 or AdEasyE1Δ2347 contained discrete DBP-containing nuclear structures that appeared as discrete foci or ring-like or reticulated structures (Figures 5A and 5B). Treatment with IFN induced little change in the formation of these structures in AdEasy E1-infected cells, whereas the majority of AdEasyE1Δ2347-infected cells exposed to the cytokine exhibited only diffuse nuclear DBP staining (Figure 5A). Quantification of the different patterns of DBP staining indicated that approximately 25% of nuclei in untreated, AdEasyE1-infected cells stained positive for DBP, but exhibited diffuse nuclear localization with no replication center formation (Figure 5B). Interferon treatment led to a less than a two-fold increase in the number of wild-type-infected cells without distinguishable replication centers. In contrast, when cells exposed to IFN were infected with AdEasyE1Δ2347, 94% of infected nuclei exhibited only diffuse DBP staining (Figure 5B).

The reduced accumulation of viral genomes and failure to form replication centers after AdEasyE1Δ2347 infection of IFN treated cells could be attributed to either a defect in *de novo* DNA synthesis, and/or rapid degradation of input DNA. To distinguish between these possibilities, viral DNA concentrations were measured in IFN treated or untreated NHBECs, as described above, between 2 and 11 hrs. p i. to monitor degradation of viral DNA early in infection. In agreement with the results described previously, IFN treatment lead to only a modest decrease in the concentration of wild-type viral DNA by 22 hrs. p. i., but a reduction of greater than 150-fold in AdEasyE1Δ2347-infected cells (Figure 5D). In untreated cells infected with 5 p.f.u./cell of either virus, modest decreases (<50% reduction) in viral DNA concentrations compared to the values measured at 2 hrs. p. i. were observed during the first few hours of infection (Figure 5C). A similar pattern was observed in IFN-treated cells, and by 11 hrs. p. i. the concentrations of intranuclear viral DNA in AdEasyE1- and AdEasyE1Δ2347-infected, IFN-treated cells were similar. These data indicate that IFN does not induce degradation of viral genomes in the absence of the E1B 55 kDa protein.

### IFN treatment does not induce apoptosis of E1B 55 kDa null-mutant-infected cells

The previously identified set of genes repressed by the E1B 55 kDa protein upon infection of normal human cells [76] includes 130 listed as interferon responsive in the Monash Institute INTERFEROME database [21] (Table S1), 15 of which are associated with apoptosis (Table 1). We therefore wished to determine if the defect in viral genome replication observed when AdEasyE1Δ2347-infected cells were treated with IFN could be attributed to induction of apoptosis, and breaks in the viral genome.

**Table 1:** Apoptosis-related genes that are induced by type I IFN and repressed by E1B 55 kDa.

Gene
CASP8 and FADD-like apoptosis regulator
Fas (TNF receptor superfamily, member 6)
interleukin 7
myeloid cell leukemia sequence 1 (BCL2-related)
myeloid differentiation primary response gene (88)
serpin peptidase inhibitor, clade B (ovalbumin), member 9
tumor necrosis factor (ligand) superfamily, member 13b
endonuclease domain containing 1
caspase 1, apoptosis-related cysteine peptidase (interleukin 1, beta, convertase)
interferon, gamma-inducible protein 16
promyelocytic leukemia; similar to promyelocytic leukemia protein isoform 1
signal transducer and activator of transcription 1, 91 kDa
toll-like receptor 2
interleukin 6 (interferon, beta 2)
interferon induced with helicase C domain 1

**Figure 6:**
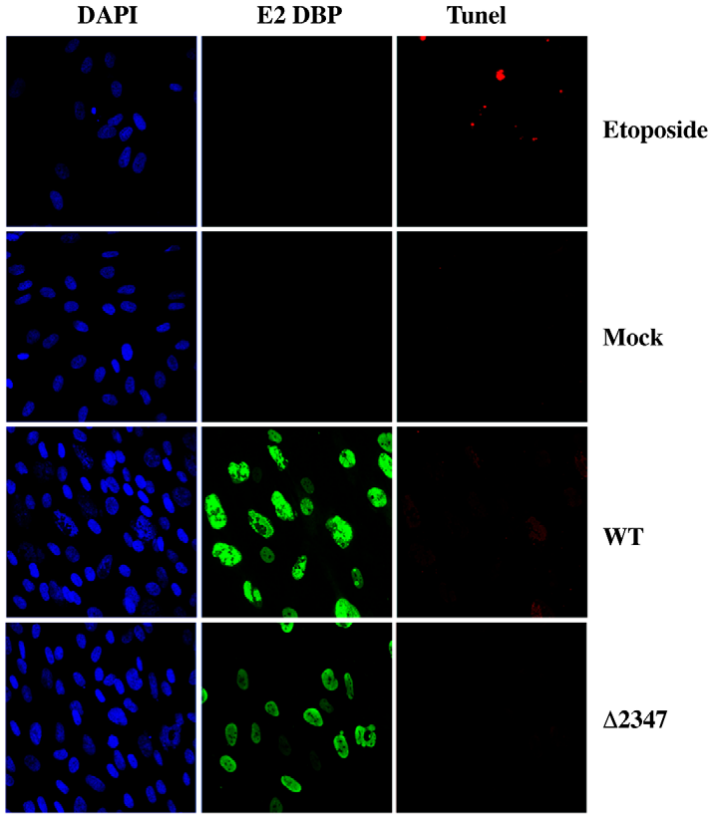
Apoptosis is not induced in FIN-treated, infected cells. IFN-treated HFFs grown on coverslips were infected with AdEasyE1 (WT) or AdEasyE1Δ2347 (Δ2347) for 34 hrs, or treated with etoposide as a positive control. Double strand breaks in DNA (red) were detected by using a TUNEL assay, as described in Materials and Methods. Immunostaining for DBP (green) was used as a marker of infection. Cells were Hoechst stained (blue), and single xy-planes visualized by confocal microscopy.

In one approach, IFN-treated and untreated HFFs were infected with AdEasyE1 or AdEasyE1Δ2347 for 34 hrs., and numbers of annexin V- and propidium iodide (PI)-positive cells measured by flow cytometry, as described in Materials and Methods, to assess induction of early apoptosis and cell death, respectively. To provide a positive control, mock-infected cells were incubated with etoposide for 34 hrs in parallel. While etoposide treatment, which induces DNA damage and apoptosis, led to a dramatic increase in the numbers of cells positive for staining with annexin V or both annexin V and PI in control experiments, no significant increase was observed in IFN treated compared to untreated cells infected with either AdEasyE1 or AdEasyE1Δ2347 (Table 2). Analysis of HFFs by TUNEL assay similarly failed to detect apoptosis in AdEasyE1 or AdEasyE1Δ2347-infected cells, regardless of whether they were exposed to IFN (Figure 6 and data not shown). These data indicate that inhibition of viral DNA synthesis in IFN treated cells infected by E1B 55 kDa null-mutants cannot be ascribed to induction of apoptosis.

**Table 2:** Interferon does not induce apoptosis in WT or E1B 55 kDa null-mutant infected cells.

Treatment^a^	% Annexin V only^b^	% Annexin V+PI^b^	% PI only^b^
Mock	1.92±0.38	1.66±0.14	2.47±0.25
Mock+Etoposide 200 uM	23.48±3.24	48.19±1.05	7.72±2.16
Mock+Etoposide 400 uM	17.80±0.04	57.34±0.57	9.70±0.39
Mock+IFN	3.12±0.56	2.44±0.64	1.96±0.31
AdEasyE1	3.16±0.49	2.07±0.02	4.10±0.38
AdEasyE1+IFN	2.25±0.03	1.61±0.02	3.21±0.01
AdEasyE1Δ2347	3.51±0.41	2.45±0.29	4.97±0.07
AdEasyE1Δ2347+IFN	4.31±0.25	1.86±0.00	2.23±0.14

a Mock or infected cells were stained and examined by FACS, as described in Materials and Methods.

b The averages and average deviations of two independent experiments are shown.

### Reorganization of cellular Pml proteins takes place normally in infected, IFN-treated cells in the absence of the E1B 55 kDa protein

**Figure 7:**
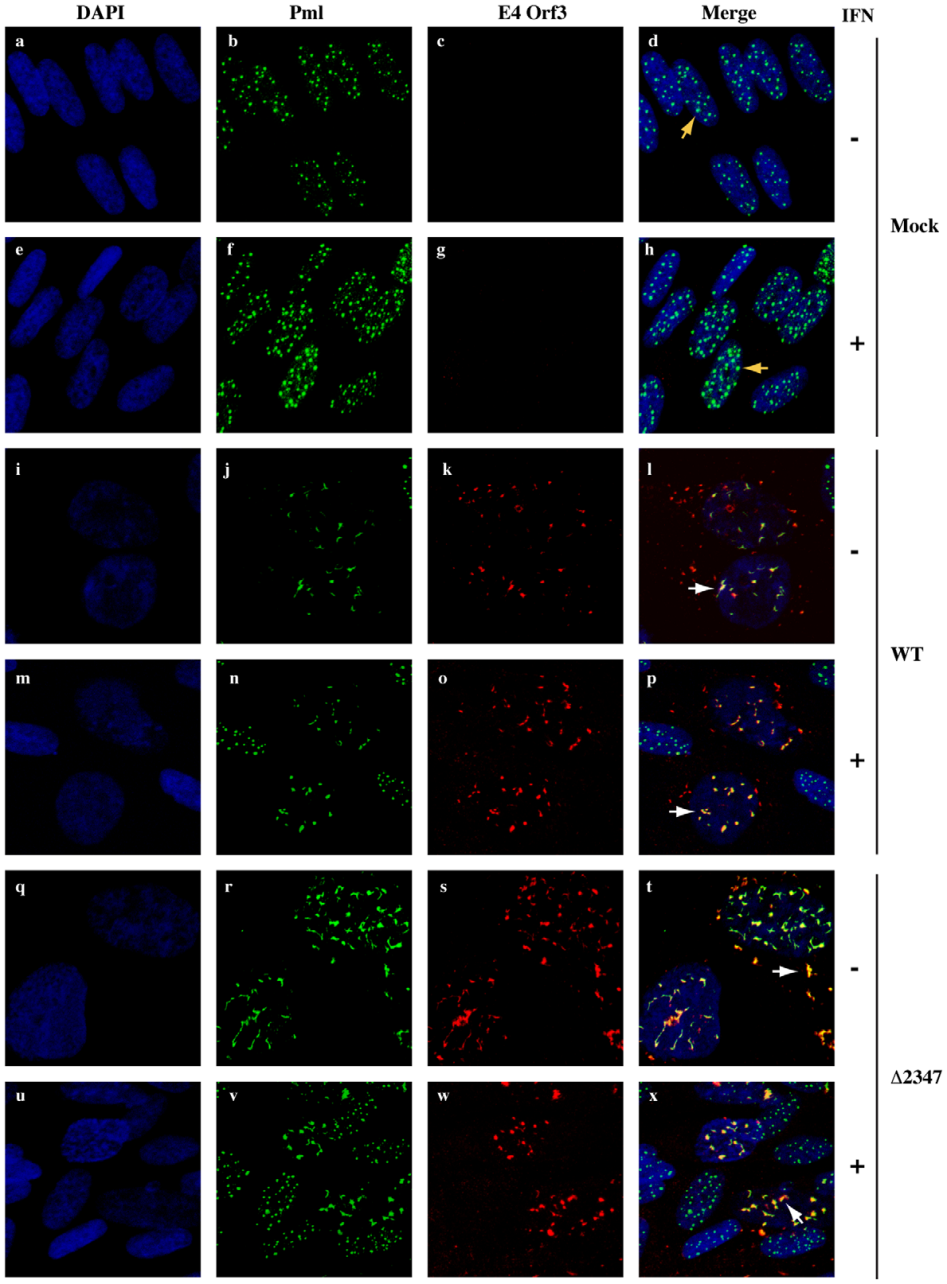
Reorganization of cellular PML proteins in AdEasyE1 and AdEasyE1Δ2347-infected, IFN-treated and untreated HFFs. HFFs grown on coverslips were IFN treated (+) or untreated (−) and infected with 30 p.f.u./cell AdEasyE1 (WT) (panels i–p) or AdEasyE1Δ2347 (Δ2347) (panels q–x), or mock infected (M) (panels a–h), and stained for the cellular Pml (green) and the adenovirus E4 Orf3 (red) proteins as described in Materials and Methods. DNA was DAPI stained (blue) and cells examined by confocal microscopy. Z-stack projections of representative fields are shown. Spherical Pml-bodies and track-like structures containing both the Pml and E4 Orf3 proteins are indicated by yellow and white arrows, respectively.

In Ad5 infected cells, nuclear Pml bodies, which are electron-dense spherical nuclear substructures defined by the presence of Pml proteins, are disrupted by the viral E4 Orf3 protein [95]–[97]. The relocalization of Pml into track-like structures that contain the E4 Orf 3 protein has been reported to be required to prevent inhibition of viral DNA synthesis induced by IFN-α or IFN-γ in Vero and IMR90 cells [32], [34]. As previous studies have indicated that the E1B 55 kDa protein associates transiently with the E4 Orf3 protein and reorganizing Pml bodies [96], [97], we wished to determine whether the E1B protein is also required for Pml relocalization in IFN-treated cells. IFN-treated or untreated HFFs were infected with 30 p.f.u./cell AdEasy E1 or AdEasy E1Δ2347, fixed at 36 hrs. p. i. and stained for Pml and E4 Orf3 as described in Materials and Methods. Mock infected, untreated cells showed Pml staining in discrete nuclear puncta numbering on average 10.4±4.8 bodies per cell (n = 80) (Figure 7, panels a–d). Exposure of mock-infected cells to IFN resulted in an increased number of Pml-staining foci of larger size (15.3±6.6 per cell, n = 80), in agreement with previous observations (see [98]). The concentration of Pml detected in wild-type infected cells was noticeably lower than that in mock-infected cells (Figure 7, compare panels b and j, and f and n), presumably because expression of the *PML* gene is repressed by the E1B 55 kDa protein [76]. As expected, the Pml protein was observed to be colocalize with the E4 Orf3 protein in distinct track-like or spherical structures (Figure 7, panels i–l, white arrows). The number of wild type-infected cells exhibiting elongated track-like structures that contained Pml and E4 Orf3 was reduced by IFN treatment, although Pml and E4 Orf3 remained colocalize (Figure 7, panels m–p). Pml bodies were also disrupted in cells infected with AdEasyE1Δ2347, exhibiting a similar distribution of Pml and E4 Orf3 signals as observed in AdEasyE1-infected cells in the presence or absence of IFN (Figure 7, panels q–x). The concentration of Pml detected in these cells was, however, higher than that observed in wild type- infected cells (Figure 7, compare panels j and r, and r and v), as expected in the absence of the E1B protein repressor of *PML* transcription. Because the relocalization of Pml proteins occurred normally in AdEasyE1Δ2347-infected cells, E1B 55 kDa must block the IFN response by a mechanism that is distinct from the disruption of nuclear Pml bodies by E4 Orf3.

### Interaction with the adenoviral E4 Orf 6 protein is not required to block the IFN-induced inhibition of viral replication

**Figure 8:**
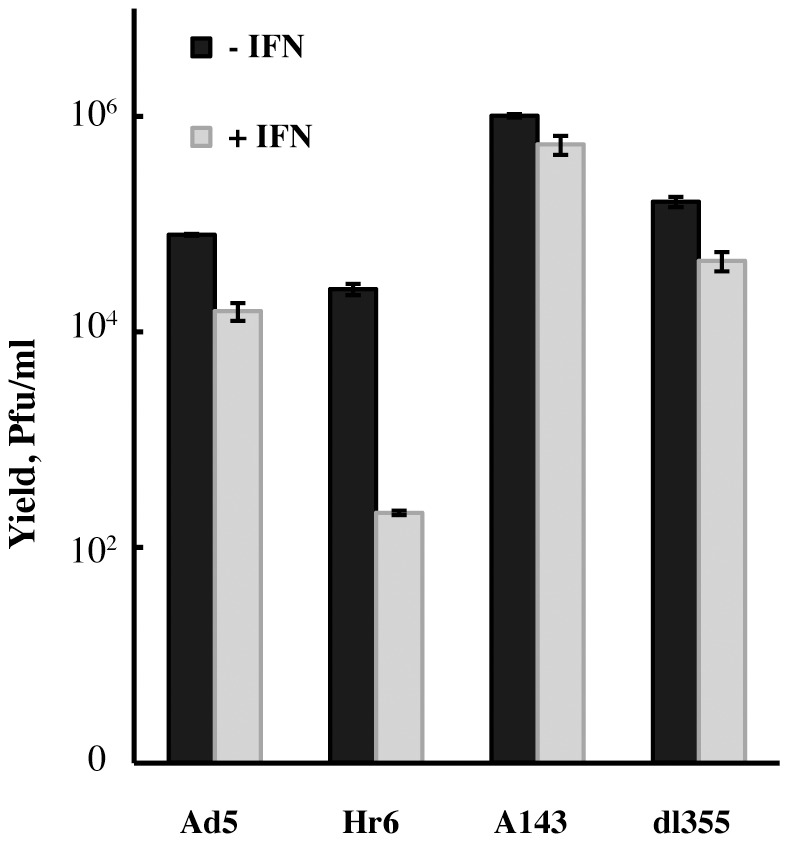
E4 Orf6 mutants that prevent assembly of the viral E3 ubiquitin ligase do not render infected cells sensitive to IFN. IFN treated and untreated NHBECs were infected with 5 p.f.u./cell Ad5, Hr6, the E4 Orf6-null-mutant dl355, or the mutant A143, in which the interaction between the E1B 55 kDa and E4 Orf6 proteins is impaired. Virus yields were determined by plaque assay 36 hours after infection. The averages and standard deviations calculated as described in Materials and Methods of two biological replicates are shown.

As discussed previously (see Introduction), assembly of a virus-specific E3 ubiquitin ligase that contains the E1B 55 kDa and E4 Orf 6 proteins is required for many of the functions fulfilled by the E1B 55 kDa protein during the infectious cycle. Furthermore, it has been reported that >95% of the nuclear E1B 55 kDa protein present during the initial period of the late phase in Ad5-infected HeLa cells is assembled into this ligase [55]. Whether this partition of the E1B 55 kDa protein is representative of other periods in the infectious cycle, such as the early phase, or of Ad5 infection of other cell types is not known. We therefore wished to determine whether the ability of the E1B 55 kDa protein to block the IFN-mediated inhibition of viral replication in normal human cells is dependent upon formation of the virus-specific E3 ubiquitin ligase. Consequently, the sensitivity to IFN of replication of the E4 Orf6-null-mutant dl355 [99] and of a mutant carrying a 4 amino acid insertion in the E1B 55 kDa protein coding sequence, A143 [100], that impairs its interaction with the E4 Orf6 protein were examined. Cells were infected with 5 p.f.u./cell of these mutants, Ad5, or the E1B 55 kDa-null-mutant Hr6, harvested at 36 hrs. p. i., and viral yields measured by plaque assay. In agreement with results described above, replication of Hr6 was inhibited to a significantly greater degree than that of Ad5 in IFN-treated cells (Figure 8). In contrast, yields of A143 and dl355 were reduced by only 1.4-fold and 2.6-fold, respectively, in cells exposed to the cytokine. These data indicate that neither the assembly of the virus specific E3 ubiquitin ligase nor the presence of the E4 Orf6 protein are required for the E1B 55 kDa protein to block inhibition of viral replication induced by IFN.

## Discussion

It is well established that the adenoviral E1B 55 kDa protein plays an important role in circumventing host cell mechanisms that limit viral replication. For example, the E1B- and E4 Orf6-protein-containing E3 ubiquitin ligase targets components of the MRN complex for proteasomal degradation [59]. When formation of this virus-specific enzyme and the ability of the E4 Orf3 protein to relocalize Mre11 are prevented by mutation, viral DNA synthesis is inhibited by a mechanism that is independent of formation of concatamers of the viral genome [62], [101]–[103]. The studies reported here establish the E1B 55 kDa protein also provides an additional, previously unrecognized defense against host anti-viral measures: a frameshift mutation that prevents synthesis of this protein renders viral replication in normal human cells sensitive to exogenous type I IFN, with reductions in virus yield of greater than two orders of magnitude (Figures 1 and 3). Three smaller related E1B 55 kDa-proteins can also be produced from E1B transcripts by alternative splicing [104]. However, the 1 bp deletion (of bp 2347 in the viral genome) that prevents production of the E1B 55 kDa protein in the null-mutants studied here [80], [82] lies downstream of the coding sequence for the N-terminal segment common to the E1B 55 kDa and its related proteins. Consequently, a function in subversion of inhibition of viral replication by IFN can be unambiguously ascribed to the E1B 55 kDa protein.

Exposure of cells to type I IFN prior to and during infection resulted in modest decreases in the accumulation of viral immediate early (E1A) and early proteins, epitomized by the E2 DBP (Figure 4A). However, the degree of inhibition of production of these proteins was the same in the absence as in the presence of the E1B 55 kDa protein (Figure 4A). This observation indicates that all prior reactions in the infectious cycle, including attachment, entry, uncoating, transport of genomes to the nucleus and initial transcription within nuclei, proceed with the same or closely similar efficiencies in IFN-treated cells infected with WT and E1B 55 kDa null-mutant viruses. In contrast, accumulation of viral genomes was observed to be strongly reduced in IFN-treated cells in the absence of the E1B protein (Figure 4B), and very few viral replication centers were formed (Figures 5A and B). At this juncture, we cannot exclude the possibility that the protection against IFN-induced mechanisms of inhibition of viral replication afforded by the E1B 55 kDa protein also extends to one or more later reactions in the infectious cycle: transcription of viral late genes requires viral DNA synthesis of infected cells [105], a reaction that is inhibited when E1B null-mutant-infected cells are exposed to IFN.

The E1B 55 kDa protein does not participate in viral DNA synthesis [105], nor is it required for this process in normal or transformed human cells [75], [80], [83]–[86], unless the onset of viral early gene expression is delayed [92], [106]. It must therefore act indirectly to allow viral genome replication and prior formation of replication centers in IFN-treated cells. The latter reaction is directed by entry of viral DNA into infected cell nuclei and does not require viral DNA synthesis. However, we found no evidence for enhanced degradation of viral DNA in IFN-treated cells when the E1B 55 kDa protein was not present (Figure 5C), consistent with the similar efficiencies of early protein synthesis observed in WT and E1B 55 kDa null-mutant-infected cells. Furthermore, induction of apoptosis, which would lead to introduction of double-stranded breaks into entering viral DNA molecules, could not be detected in either WT- or E1B 55 kDa null-mutant-infected cells exposed to type 1 IFN (Figure 6, Table 2), despite the repression of transcription of several apoptosis-associated ISGs by the E1B protein (Table 1). *In toto*, these observations suggest that formation of viral replication centers and genome replication depend on one or more alterations in intranuclear structures or components that can take place in IFN-treated cells only when the E1B 55 kDa protein is made.

Several viruses with DNA genomes that are replicated in infected cell nuclei, including polyomaviruses, herpesviruses and adenoviruses, encode proteins that disrupt the intranuclear structures termed Pml bodies (a.k.a. ND IOs), and in this way are thought to circumvent an intrinsic anti-viral defense [98], [107], [108]. In the case of Ad5, the E4 Orf3 protein sequesters Pml in distinctive track-like structures [95]–[97]. Such relocalization of Pml is dispensable for viral replication, at least in established lines of human cells, as mutations that prevent synthesis of the E4 Orf3 protein, or its interaction with Pml, exhibit no growth defects following high multiplicity infection [109], [110]. However, this reaction is necessary for efficient formation of viral replication centers in normal diploid fibroblasts or Vero cells exposed to IFN α [32], [34], which induces increased transcription of the genes that encode Pml and other proteins present in Pml bodies [98]. Inhibition of synthesis of Pml or the Pml body-associated co-repressor Daxx by RNAi restored formation of replication centers in E4 Orf3 mutant-infected cells exposed to exogenous IFNα, and the inhibitory effects of Daxx were shown to be independent of effects on expression or functions of viral early gene products. These observations establish a critical role for Pml bodies in IFN-induced inhibition of adenoviral replication. Although the mechanism of action of Pml body components remains unclear, Hearing and colleagues proposed that Pml and Daxx might function together to inhibit viral DNA synthesis in IFN-treated cells, or that Daxx functions as the effector of such inhibition upon Pml-dependent recruitment to Pml bodies [34]. The E1B 55 kDa protein has been reported to interact with both the E4 Orf3 protein during the initial period of the early phase of infection [97] and with Pml-bodies [96]. Furthermore, the defects in formation of viral replication-centers in IFN-treated cells observed in the absence of the E4 Orf3 [32] or the E1B 55 kDa (Figures 5A, B) proteins are very similar. Nevertheless, the E4 Orf3 protein sequesters Pml efficiently, even when this cellular protein is overproduced in IFN-treated cells in the absence of the E1B 55 kDa protein (Figure 7). This observation indicates that the E1B protein is dispensable for the protective reorganization of Pml bodies by the E4 Orf3 protein, and hence that these two viral early proteins block inhibitory effects of IFN by different mechanisms.

The E1B 55 kDa protein has been reported to repress transcription of p53-regulated genes via binding to p53 and to contain a repression domain that inhibits transcription of reporter genes when fused to an heterologous DNA-binding domain [67], [69], [70]. This protein also induces inhibition of export of mature cellular mRNAs from the nucleus to the cytoplasm [80], [86]. As such cellular mRNAs do not accumulate in infected cell nuclei [111], this activity could contribute to repression of expression of interferon-sensitive genes by the E1B 55 kDa protein [76]. However, regulation of mRNA export in infected cells depends on assembly of the E1B- and E4 Orf6 protein-containing E3 ubiquitin ligase [64], [65], whereas protection of viral replication from IFN-induced inhibition does not (Figure 8). Rather, the E1B 55 kDa protein blocks accumulation of the primary transcripts of several ISGs in normal human cells, either untreated or exposed to exogenous IFN (Figure 2). It remains possible that the E1B 55 kDa protein inhibits splicing of such pre-mRNAs, or promotes their intranuclear turnover. However, this viral protein has not been implicated in regulation of pre-mRNA processing. Furthermore, we have observed recently that substitutions within the previously identified repression domain of the E1B 55 kDa protein [69] impair inhibition of synthesis of ISG pre-mRNAs in normal human cells (J.S.C., C. Gallagher and S.J. F., manuscript in preparation). These observations are consistent with the conclusion that the E1B 55 kDa protein represses transcription of specific cellular genes during the productive cycle, and identify ISGs as an important target. We therefore propose that this viral protein permits viral DNA synthesis in IFN-treated cells by repressing transcription of one or more specific ISGs that encode a protein that either prevents formation of viral replication centers, or removes, or inactivates protein(s) essential for this process. An important implication of this hypothesis is that formation of replication centers is an active process essential for successful viral DNA synthesis and replication, rather than simply the result of association of viral DNA molecules and replication proteins within infected cell nuclei. It will therefore be of considerable interest to identify the cellular protein(s) that is targeted by the E1B 55 kDa protein to facilitate this reaction in the infectious cycle in IFN-treated cells. Although intranuclear sites of viral genome replication and transcription have been characterized in some detail [112], very little is known about either the initial intranuclear localization and molecular interactions of Ad5 DNA, or dynamic changes that might be required for formation of viral replication centers. Furthermore, none of the host protein previously reported to be associated with viral replication centers, Sp100 [96], Mdc1 [63], and several proteins that participate in Atr-dependent signaling (Atr itself, Atrip, Rpa32, TopBP1 and E1B-Ap5/Hnrl1) [113]–[115] appear to be likely candidates: only the Pml body component Sp100 is encoded by an IFN-inducible gene [98] that is transcriptionally repressed by the E1B 55 kDa protein [76], and, as discussed above, disruption of Pml bodies by the E4 Orf3 protein takes place normally in IFN-treated cells infected by the E1B 55 kDa null-mutant virus.

As noted previously, it is well established that the E1B 55 kDa protein can act as a direct repressor of transcription in simplified experimental systems [66], [67]. The mechanism of such repression remains incompletely understood, but has been proposed to reflect the recruitment of co-repressors to promoters by the viral protein [116]. The E1B 55 kDa protein has indeed been reported to interact with the cellular repressors Daxx [117] and via Sin3a, HdacI [118], [119], but the physiological consequences of these interactions have not been elucidated. At this juncture, it is not clear that this viral protein functions as a direct repressor of ISG transcription in infected cells. The virus-specific E3 ubiquitin ligase that contains the E1B 55 kDa protein is not required to prevent inhibition of viral replication by IFN (Figure 8). However, the E1B protein can also function as a SUMO-1 E3 ligase independently of the E4 Orf6 protein [119], [120], and could therefore modify and inactivate transcriptional regulators necessary for efficient transcription of IFN-inducible genes. Experiments are in progress to investigate whether the E1B kDa protein directly or indirectly represses transcription of cellular genes during the infectious cycle.

The discovery of the previously unrecognized participation of the E1B 55 kDa protein in obstructing inhibition of viral replication by IFN indicates that at least four viral gene products help counter this host defense. Such a multiplicity is not unexpected, as many viruses that replicate in mammalian cells circumvent the anti-viral actions of IFN via multiple mechanisms and gene products [22], [24], [94]. The contributions of the Ad5 E1A, E1B 55 kDa and E4 Orf3 proteins and VA-RNA I to blocking inhibition of replication by IFN have not been evaluated systematically. However, the information currently available argues that these viral gene products function by different, non-redundant mechanisms. The E4 Orf3 protein and VA RNA I block the inhibitory effects of products of IFN-inducible genes, namely Pml [34] and Pkr (aka Eif2ak2) [33], respectively. In contrast, E1A proteins act prior to transcription of such genes by impairing assembly of the critical transcriptional activator [38], [40]. Like E1A proteins, the E1B 55 kDa protein likely represses transcription of IFN-inducible genes. However, this protein also inhibits expression of several genes encoding proteins that induce synthesis of IFN in response to viral infection, including Rig 1 (aka Ddx58), Mda5 (aka If1h1), Irf7 and Myd88 [76], suggesting that it also blocks the initial production of anti-viral cytokines. Experiments are in progress to test the hypothesis that the E1B 55 kDa protein is a broadly acting inhibitor of induction of the IFN response that complements the mechanism of E1A protein-mediated inhibition.

The finding that the E1B 55 kDa protein is a potent inhibitor of induction of the IFN-mediated anti-viral defenses in Ad5-infected normal human cells is clearly of interest in the context of development of adenoviral vectors. It is likely that the deletion of the coding sequence for this E1B protein common to such vectors contributes to the induction of vigorous innate immune responses *in vivo*, particularly upon systemic delivery (see Introduction). Typical vectors lacking E1A, E1B and E3 genes have been reported to induce increased expression of many genes in the liver of C57/B16 mice [12], including genes repressed by the E1B 55 kDa protein in normal human cells [76]. However, it will be important both to identify genes repressed by the E1B 55 kDa protein and to investigate the role of this protein in blocking innate responses to infection *in vivo*. The identification of specific mutations that eliminate the transcriptional repression function of this E1B protein, but confer potentially advantageous properties such as tumor-cell selective replication, may also facilitate the design of more efficacious adenoviral vectors. Alternatively, this function may help account for the poorly understood tumor cell-selective replication of first generation oncolytic adenoviruses [88], which carry mutations that prevent synthesis of the E1B 55 kDa and related proteins [105]: many human tumor cells carry mutations that result in defects in the production or response to IFN [121]–[123].

## Materials and Methods

### Cells and viruses

293 cells were maintained in Dulbecco's modified Eagle's medium (DMEM, GIBCO) containing 5% bovine growth serum (Thermo Scientific Hyclone) and 5% calf serum (GIBCO). Human foreskin fibroblasts (HFFs) were maintained in DMEM containing 7.5% bovine growth serum. Primary human bronchial/tracheal epithelial cells (NHBECs) were obtained from BioWhittaker Inc. and maintained in bronchial epithelial growth media (BEGM, Lonza), and passaged according to the manufacturer's recommendations. Universal Type I Interferon was obtained from PBL InterferonSource and diluted in sterile PBS containing 0.1% (w/v) BSA. Cells were pretreated with the indicated concentration of IFN or vehicle only (PBS plus 0.1% BSA) for 24 hrs. prior to infection. The construction of a phenotypically wild-type derivative of AdEasy [81] containing the E1A and E1B genes (AdEasyE1) and the introduction of the Δ2347 mutation into this background to create AdEasyE1Δ2347 have been described [82]. These viruses, Ad5 and the E1B 55 kDa-null-mutant Hr6 [79] were propagated and titered in 293 cells. To monitor viral replication, cells at <90% confluency were infected for the periods indicated, harvested and washed once with PBS, and cell pellets resuspended in 0.01 M Tris–HCl, pH 7.4, containing 0.15 NaCl, 0.005 M KCl, 10 mM MgCl_2_, and 0.1% (w/v) dextrose. Samples were freeze-thawed 4 times and debris removed by centrifugation at 13000×g for 5 minutes at 4°C. Concentrations of infectious particles units (P.f.u.s) were measured by plaque assay on complementing 293 cells as described [124]. Plaque assays were performed at least in triplicate, and standard deviations plotted for each data point indicate the combined propagated standard deviations of these assays and biological replicates of the experiments.

### Analysis of cellular mRNAs and pre-mRNAs

Virus- or mock-infected cells were harvested 24 hrs. (NHBECs) or 30 hrs. (HFFs) after infection, washed once with PBS, and lysed in 20 mM Tris pH 7.5, containing 2 mM EDTA, 0.15 M NaCl, and 0.65% (v/v) NP-40. NaCl was added to a final concentration of 0.5 M and samples diluted with 0.6 volumes water pre-treated with diethyl pyrocarbonate. One volume of 2× proteinase K buffer (20 mM Tris pH 7.5, containing 0.15 M NaCl, 2% (w/v) SDS, 2 mM EDTA), and 200 µg proteinase K (NEB) were then added, and samples incubated for 30 min at 37°C. Samples were extracted with (1∶1) phenol-CHCl_3_ and ethanol precipitated. Following resuspension in DNase I digestion buffer (Roche), solutions were incubated with 10 U DNase I (Roche) at 37°C for 30 min prior to phenol:CHCl_3_ extraction and ethanol precipitation. RNA samples were resuspended in 10 mM Tris, pH 7.5, containing 5 mM NaCl, and 0.5 U/µl RNasin (Promega). RNA concentrations were determined from A260 reading made using a NanoDrop ND-1000 spectrophotometer. cDNA was synthesized from 1 µg of RNA by priming with 200 ng random hexamers (Roche) and extension with SuperScript II reverse transcriptase (Invitrogen) using the conditions recommended by the manufacturer. ISGs were detected by PCR with the following primers (5′ to 3′) and reaction conditions: IFIT2 primary transcript, fwd: GAGTGCAGCTGCCTGAACCGAGCC, rev: GCAACTCAACTCCCCCAGGCGTGC, 60.9°C annealing, 67°C extension, 32 cycles; IL6 primary transcript, fwd: GCCCACCGGGAACGAAAGAGAGC, rev: CCTGGGCCACACACCCCTCC, 59°C annealing, 66°C extension, 32 cycles; STAT1 primary transcript, fwd: CTCGACAGTCTTGGCACCTAACG, rev: CATTAAGCCCTTCCATCTTTGAACATA, 53°C annealing, 60°C extension, 25 cycles; GBP1 mRNA, fwd: GTCAACGGGCCTCGTCTAGA, rev: CCCACTGCTGATGGCAATG, 50°C annealing, 65°C extension, 30 cycles; GAPDH mRNA transcript, fwd: CTGTTGCTGTAGCCAAATTCGT, rev: ACCCACTCCACCTTTGAC, 50°C annealing, 65°C extension, 20 cycles. PCR products were resolved by electrophoresis in 8% polyacrylamide gels. Signals were quantified using Image J.

### Measurement of viral DNA concentrations

NHBECs in 6-well dishes were infected with 5 p.f.u./cell AdEasyE1or AdEasy E1Δ2347 and harvested after the periods of infection indicated. DNA was isolated from nuclei as previously described [106]. Quantitative real-time PCR was carried out using the ABI PRISM 7900HT sequence detection system with SYBR Green Master Mix (Applied Biosystems) to detect an amplicon within the ML transcription units, 90 base pairs long (nucleotides 7128 to 7218). The primers used were as follows: fwd: ACT CTT CGC GGT TCC AGT ACT C, rev: CAG GCC GTC ACC CAG TTC TAC. 20 µl reactions contained 2 µl sample DNA, diluted 1∶100 for ML amplification, and undiluted for the detection of genomic β-actin DNA as an internal cellular control, using the primers: fwd: TCCTCCTGAGCGCAAGTACTC, rev: ACTCGTCATACTCCTGCTT. Experiments were carried out in biological duplicate. PCR cycles were programmed as follows: two initial steps at 50°C for 2 min and 95°C for 10 min, and then 40 cycles of 95°C for 15 sec and 60°C for 60 sec. Relative DNA concentrations were determined by the standard curve method using the plasmid pTG3602, which contains the Ad5 genome sequence, as reference standard for ML detection, and a recombinant HCMV BAC containing the genomic human β-actin sequence, (a kind gift of Thomas Shenk) as standard for the internal control. All qPCR measurements were performed in triplicate. Mean ML values were corrected with respect to the mean β-actin values for each sample before normalization to the 2 hrs. p. i. input value. Standard deviations plotted for each data point represent the combined propagated standard deviations of the qPCR assay and biological replicates of the experiment.

### Immunoblotting

NHBECs at 80–90% confluence were infected with AdEasyE1or AdEasy E1Δ2347 as described above. Cells were harvested at the times after infection indicated, washed with phosphate-buffered saline (PBS), and extracted with 25 mM Tris-HCl, pH 8.0, containing 50 mM NaCl, 0.5% (w/v) sodium deoxycholate, 0.5% (v/v) Nonidet P-40 (NP-40) and 1 mM phenylmethylsulfonyl fluoride for 30 min at 4°C. Extracts were sonicated for a total of 30 s or incubated with 125 units Benzonase nuclease (Sigma) for 30 minutes at 37°C, and cell debris removed by centrifugation at 10,000×g for 5 min at 4°C. The extracts were analyzed by sodium dodecyl sulfate (SDS)-polyacrylamide gel electrophoresis and immunoblotting as described previously [125]. The E1A and E2 DBP proteins were detected with the monoclonal antibodies M73 [126], and B6 [127] respectively. β-actin was examined using a horseradish peroxidase-conjugated monoclonal antibody (Abcam) to provide an internal loading control.

### Immunoflourescence

HFFs grown to no more than 90% confluence on sterile coverslips were mock infected, or infected with AdEasyE1or AdEasy E1Δ2347 for 36 hrs., and the cells processed for immunoflourescence as described previously [125]. To examine replication centers, the viral E2 DBP was visualized using the B6 antibody [127] and Alexa 488 anti-mouse IgG (Invitrogen), and DNA was stained with DAPI (Invitrogen). Coverslips were mounted on glass slides in Aqua Polymount (Polysciences Inc.) and images acquired using a Zeiss Axiovert 200 M fluorescence microscope and AxioVision software. Cellular Pml proteins were detected using the monoclonal antibody PAB14682 (Abnova), with Alexa 488 anti-rabbit IgG (Invitrogen), and the E4 Orf3 protein by using the rat monoclonal antibody 6A11 [128] and Alexa 568-conjugated goat anti-rat IgG (Invitrogen). Coverslips were mounted as described above, and samples examined by confocal microscopy using a Zeiss LSM 510 confocal system. All images were organized using Adobe Photoshop 7.0.

### Assays for apoptosis

HFFs grown to 80% confluency in 6-well dishes were pretreated with 500 U/ml IFN or BSA-only for 12 hrs prior to mock infection or infection for 34 hrs with 200 p.f.u./ml of the viruses indicated. IFN treatment was resumed after adsorption. To provide a positive control, HFFs exposed to 200 µM etoposide for 34 hrs. were included in the analysis. Medium was collected and pooled with trypsinized cells. Cells were pelleted by centrifugation at 16,000×g for 3 min, washed once with PBS, and resuspended in 0.5 ml 10 mM Hepes-NaOH, pH 7.4, containing, 0.14 M NaCl, and 2.5 mM CaCl_2_ (binding buffer). AlexaFluor 488-conjugated annexin V (1∶20 final dilution) (InVitrogen), and propidium iodide (5 µg/ml final concentration) was added to 100 µl aliquots of the cell suspensions, and incubated for 15 min at room temperature. Volumes were brought up to 500 µl with binding buffer, and samples were analyzed by flow cytometry using a BD LSRII Multi-Laser Analyzer. Experiments were carried out in biological duplicate. Terminal deoxynucleotidyl transferase dUTP nick end labeling (TUNEL) assays were performed using a Click-iT TUNEL AlexaFluor 488 Imaging Assay kit (Invitrogen). HFFs grown on coverslips were pretreated with 500 U/ml IFN for 12 hrs and were mock-infected or infected for 34 hrs. with 200 p.f.u./ml of the virus indicated, and IFN treatment resumed after adsorption. As a positive control, uninfected HFFs were treated for 34 hrs. with 200 µM etoposide. Cells were fixed, and TUNEL reactions and staining were performed exactly according to the manufacturer's protocol. DBP and DNA staining (Hoechst, Invitrogen) were performed after TUNEL as described above, except that the secondary antibody used to visualize DBP was AlexaFluor 555-conjugated anti-mouse IgG (Invitrogen).

## Supporting Information

Table S1 **Interferon-inducible genes repressed by the E1B 55 kDa protein.** Shown are previously identified interferon-inducible human genes that were observed to be increased in expression in normal human fibroblasts infected by the E1B 55 kDa-null mutant Hr6 compared to cells infected by Ad5 (76).
(XLSX)
